# Supplementary material for: Organizational debt—Roadblock to agility in software engineering: Exploring an emerging concept and future research for software excellence
Source: PLoS One. 2024 Nov 25;19(11):e0308183. doi: 10.1371/journal.pone.0308183 (PMC11588203; doi:10.1371/journal.pone.0308183)
Supplement: Attachment — Submitted filename: Response to Reviewers.docx [file pone.0308183.s002.docx]

Dear Editors and Reviewers,

Thank you for your valuable feedback and suggestions on our manuscript titled "Organizational Debt in Software Engineering: A Multivocal Literature Review." We appreciate the time and effort you have invested in reviewing our work and providing constructive comments to improve its quality.

We have carefully considered the points raised by the reviewer and would like to address them in this response letter.

Regarding the concern about the lack of data to sufficiently ground the findings, we acknowledge the limitation of relying solely on blog posts and practitioner literature. As mentioned in the paper, the topic of organizational debt (OD) is an emerging concept in software engineering, and there is a scarcity of academic literature on this subject. Our motivation for conducting a multivocal literature review (MLR) was to synthesize the perspectives and experiences of industry professionals, who are at the forefront of grappling with OD challenges.

However, we recognize the importance of complementing these practitioner insights with academic research to strengthen the validity and rigor of our findings. We thank the reviewer for suggesting the paper by William F. Jarvis and the case study by Zixuan Liu et al. These sources provide valuable insights from the management and organization domains, which can enrich our understanding of OD in the software engineering context.

**To address this concern, we propose the following revisions to our manuscript:**

1. **We conducted a thorough search for relevant academic literature on OD from related disciplines**, such as management, organizational behavior, and information systems. This has broaden our data corpus and has provided a more comprehensive view of the phenomenon. **Please, see pages 4-6**

**2. We incorporated the suggested papers by William F. Jarvis and Zixuan Liu et al., as well as eight (8) other relevant academic sources, into our analysis**. This allows us to triangulate the practitioner perspectives with theoretical and empirical findings from academic research. **Please, see results section on pages 5-6.**

3. In the revised manuscript, **we clearly distinguish between insights derived from practitioner literature and those from academic sources**. This will hopefuly enhance transparency and will allow the reader to assess the credibility and generalizability of our findings. **Please, see results section on pages 5-6.**

4. **We adjusted the methodology section** to reflect the inclusion of academic literature and provide a detailed description of the search strategy, selection criteria, and analysis process for these additional sources. **Please, see research method section on pages 4-5.**

**5. The discussion section has been expanded to critically evaluate the convergence or divergence of practitioner and academic perspectives on OD**, as well as the implications for software engineering research and practice. **Please, see discussion section on pages 13-14.**

We believe that addressing the reviewer's concern by incorporating relevant academic literature has strengthen our study's validity and contribute to a more comprehensive understanding of the organizational debt phenomenon in software engineering.

Thank you again for your valuable feedback and the opportunity to improve our work. We look forward to addressing these concerns and submitting a revised manuscript for your consideration.

Sincerely,

On behalf of the authors,

Osama Al-Baik
